# Airborne fungal spores and invasive aspergillosis in hematologic units in a tertiary hospital during construction: a prospective cohort study

**DOI:** 10.1186/s13756-019-0543-1

**Published:** 2019-05-29

**Authors:** Joung Ha Park, Seung Hee Ryu, Jeong Young Lee, Hyeon Jeong Kim, Sun Hee Kwak, Jiwon Jung, Jina Lee, Heungsup Sung, Sung-Han Kim

**Affiliations:** 1 Department of Infectious Diseases, Asan Medical Center, University of Ulsan College of Medicine, 88, Olympic-ro-43-gil, Songpa-gu, Seoul, 05505 Republic of Korea; 2 Office for Infection Control, Asan Medical Center, University of Ulsan College of Medicine, Seoul, Republic of Korea; 3 Department of Pediatrics, Asan Medical Center, University of Ulsan College of Medicine, Seoul, Republic of Korea; 4 Department of Laboratory Medicine, Asan Medical Center, University of Ulsan College of Medicine, Seoul, Republic of Korea

**Keywords:** Fungal spore, Invasive aspergillosis, Construction

## Abstract

**Background:**

Invasive aspergillosis (IA) is an opportunistic fungal infection that mostly occurs in immunocompromised patients, such as those having hematologic malignancy or receiving hematopoietic stem cell transplantation. Inhalation of *Aspergillus* spores is the main transmission route of IA in immunocompromised patients. Construction work in hospitals is a risk factor for environmental fungal contamination. We measured airborne fungal contamination and the incidence of IA among immunocompromised patients, and evaluated their correlation with different types of construction works.

**Methods:**

Our tertiary hospital in Seoul, Korea underwent facility construction from September 2017 to February 2018. We divided the entire construction period into period 1 (heavier works: demolition and excavation) and period 2 (lighter works: framing, interior designing, plumbing, and finishing). We conducted monthly air sampling for environmental spore surveillance in three hematologic wards. We evaluated the incidence of IA among all immunocompromised patients hospitalized in the three hematologic wards (2 adult wards and 1 pediatric ward) during this period. IA was categorized into proven, probable, and possible aspergillosis based on the revised European Organization for Research and Treatment of Cancer/Mycosis Study Group (EORTC/MSG) criteria.

**Results:**

A total of 15 patients was diagnosed with proven (1 case), probable (8 cases), or possible (6 cases) hospital-acquired IA during period 1. In period 2, 14 patients were diagnosed with either proven (1 case), probable (10 cases), or possible (3 cases) hospital-acquired IA. Total mold and *Aspergillus* spp. spore levels in the air tended to be higher in period 1 (*p* = 0.06 and 0.48, respectively). The incidence rate of all IA by the EORTC/MSG criteria was significantly higher in period 1 than in period 2 (1.891 vs. 0.930 per 1000 person-days, *p* = 0.05).

**Conclusions:**

Airborne fungal spore levels tended to be higher during the period with heavier construction works involving demolition and excavation, during which the incidence of IA was significantly higher as well. We recommend monitoring airborne fungal spore levels during construction periods in hospitals with immunocompromised patients. Subsequently, the effect of airborne fungal spore level monitoring in reducing hospital-acquired IA should be evaluated.

**Electronic supplementary material:**

The online version of this article (10.1186/s13756-019-0543-1) contains supplementary material, which is available to authorized users.

## Background

Invasive aspergillosis (IA) is an opportunistic fungal infection that mostly occurs in immunocompromised patients, such as those having hematologic malignancy or receiving hematopoietic stem cell transplantation [1]. The treatment of IA is difficult and the mortality rate is reported as 30–58%, especially among immunocompromised patients [2–4]. Inhalation of *Aspergillus* spores, which are ubiquitously found in soil, water, and air, is the main transmission route of IA [5]. Accordingly, there have been many efforts to control environmental fungal contamination using appropriate ventilation systems, such as high-efficiency particulate air (HEPA) filters [6–8].

Construction works in hospitals are risk factors for environmental fungal contamination and hospital-acquired IA [8–11]. In a previous review, construction works accounted for about half (49%) of the causes of hospital-acquired aspergillosis outbreaks [11]. It is recommended that clinicians conduct active surveillance for airborne fungal infection in high-risk patients and that wards for these patients should be equipped with appropriate ventilation systems based on infection-control risk assessment during construction periods [6]. However, it remains controversial whether elevated fungal spore level assessed by air sampling during construction works in hospitals is associated with the increased risk of IA [10, 12, 13], although it is well known that construction works in hospitals have been associated with IA outbreaks [8, 10, 11]. Therefore, the recommendation of routine microbiologic air sampling before, during, or after construction is still under debate. We thus evaluated the correlation between airborne fungal contamination and the incidence of IA among immunocompromised patients with hematologic malignancy during hospital construction periods. We also investigated fungal spore levels in the air and the incidence of IA depending on the types of construction.

## Methods

### Study design

This study was conducted at Asan Medical Center, a 2700-bed tertiary care teaching hospital in Seoul, South Korea. A radiotherapy facility construction was carried out behind the main buildings from September 2017 to February 2018. We included patients with hematologic malignancies who were hospitalized in three hematologic wards during this period. The three hematologic wards consist of two adult wards (wards 74 and 84) and one pediatric ward (ward 146). Ward 74 is composed of 16 rooms, eight of which are dedicated for hematopoietic stem cell transplantation (HSCT) and are equipped with HEPA filters and positive pressure ventilation systems; the remaining eight conventional rooms do not have specific ventilation systems. Ward 84 is composed of conventional rooms only. The pediatric ward 146 is composed of rooms, seven of which are dedicated to HSCT and equipped with both HEPA filters and positive pressure ventilation systems; the remaining rooms are equipped with HEPA filters only. The three wards are all isolated from the other general wards by a door at the ward entrance. During the construction periods, all windows were recommended to be kept closed. We divided the entire construction period into two periods—period 1 (September–October) with heavier works such as demolition and excavation, and period 2 (November–February) with lighter works such as framing, interior designing, plumbing, and finishing. We measured airborne fungal contamination by air sampling and compared the incidence of IA in the three wards between periods 1 and 2. Posaconazole was used as antifungal prophylaxis in patients who underwent induction chemotherapy for acute myeloid leukemia and myelodysplastic syndrome or hematopoietic stem cell transplant recipients with graft versus host disease. Micafungin was used as antifungal prophylaxis in hematopoietic stem cell transplant recipients. Antifungal prophylaxis was same during period 1 and period 2. The study protocol was approved by the Institutional Review Board of Asan Medical Center.

### Definition

IA was categorized into proven (histopathologic evidence of tissue invasion including septated, acutely branching filamentous fungi, or positive culture from sterile specimens), probable (presence of a host factor, a clinical criterion, and a mycological criterion compatible with IA), and possible (presence of a host factor and a clinical criterion without mycological evidence) aspergillosis based on the revised European Organization for Research and Treatment of Cancer/Invasive Fungal Infections Cooperative Group and the National Institute of Allergy and Infectious Diseases Mycoses Study Group (EORTC/MSG) criteria [14]. To identify patients with IA, a researcher (JH Park) retrospectively reviewed the demographic characteristics, clinical manifestations, laboratory findings, and imaging studies of all suspected IA patients based on electronic medical records. The results of air sampling and the study period were concealed from the researcher to avoid bias. Patients who were diagnosed with IA using the aforementioned criteria were finally included in this study. We only analyzed cases of patients who were presumptively classified as having hospital-acquired IA, defined as those diagnosed with IA at least one week after admission [15] and had no clinical or radiological signs of IA at admission.

### Air sampling for environmental surveillance

Air sampling for environmental surveillance was conducted once a month in the three hematologic wards during the construction periods. Airborne fungal contamination was determined by the total mold spore concentration and the level of *Aspergillus* spp. spores in air. We collected 1000 L of air three times every 20 min by using a portable air sampler (AirPort MD8, Sartorius AG, Germany) located at each nurse station in the three hematologic wards. Air was plated onto Sabouraud dextrose agar and incubated at 30 °C for five days. After incubation, we counted colonies and expressed the data as median colony-forming units (CFU) per 1000 L of air. Colonies were identified at the genus level based on macroscopic and microscopic findings (lactophenol cotton blue-stained preparation). Finally, quantitative results were generated for both *Aspergillus* spp. and total molds.

### Statistical analysis

In comparing the clinical characteristics of patients between periods 1 and 2, categorical variables were compared using the *χ*^2^ or Fisher’s exact test as appropriate, and continuous variables were compared using the Mann-Whitney*U* test. We assessed *P* for trend of total mold and *Aspergillus* spp. spores using a linear regression model. Also, we compared the incidence of IA between the two periods using a Poisson regression model. All tests of significance were two-tailed and *p* values ≤0.05 were considered statistically significant. All analyses were performed using SPSS for Windows, version 21.0 (IBM Corp., Armonk, NY, USA).

## Results

### Study population

The clinical characteristics of patients with IA are shown in Table 1. A total of 15 patients was diagnosed with either proven (1 case), probable (8 cases), or possible (6 cases) hospital-acquired IA during period 1. In period 2, 14 patients were diagnosed with either proven (1 case), probable (10 cases), or possible (3 cases) hospital-acquired IA. In the entire construction period, four and three pediatric patients were included in periods 1 and 2, respectively. Of a total of 29 IA patients, two (1 in period 1 and the other in period 2) were diagnosed with proven invasive *Aspergillus* sinusitis, and the remaining patients were all diagnosed with invasive pulmonary aspergillosis. These two patients with invasive fungal sinusitis, not invasive pulmonary aspergillosis, had undergone paranasal sinus magnetic resonance imaging. The remaining 27 patients had all undergone chest computed tomography scan at hospitalization. Acute myeloid leukemia was the most common underlying hematologic disease (47% vs. 33%) in both periods. Neutropenia was common in both periods (82% vs. 80%) and patients who underwent HSCT accounted for 41 and 33% in periods 1 and 2, respectively. Patients were usually treated with voriconazole or amphotericin B. Four patients in each period had microbiologically confirmed IA by a fungal culture of respiratory specimens or biopsy tissues; four yielded *Aspergillus fumigatus*, three yielded *Aspergillus flavus*, and one yielded *Aspergillus niger*. Of these patients, seven tested positive for serum galactomannan, and the remaining patient with invasive *Aspergillus* sinusitis tested negative for serum galactomannan.

**Table 1:** Clinical characteristics of patients with invasive aspergillosis at the time of IA diagnosis

	Period 1^a^ (*n* = 15)	Period 2^b^ (*n* = 14)
Age, median years (IQR)	44 (17–60)	59 (43–63)
Male gender, no. (%)	10 (59)	11 (73)
Underlying hematologic disease, no. (%)		
Acute myeloid leukemia	8 (47)	5 (33)
Acute lymphoid leukemia	1 (6)	2 (13)
Myelodysplastic syndrome	3 (18)	0 (0)
Lymphoma	2 (12)	1 (7)
Others^c^	3 (18)	7 (47)
Immunocompromised condition		
Neutropenia^d^	12 (80)	11 (79)
Hematopoietic stem cell transplant	6 (40)	5 (36)
Steroid use^e^	3 (20)	3 (21)
T-cell immunosuppressant	2 (13)	3 (21)
Human immunodeficiency virus infection	1/15^f^ (7)	0/12^f^ (0)
Symptoms at the time of IA diagnosis		
Fever	13 (87)	11 (79)
Cough	4 (27)	4 (29)
Sputum	2 (13)	4 (29)
Dyspnea	6 (40)	3 (21)
Positive results for serum galactomannan	9 (53)	12 (80)
Positive results for beta-D-glucan	8/14^f^ (57)	9/12^f^ (75)
Positive results for culture specimens	4 (24)	4 (27)
Hospitalization days before IA diagnosis, median days (IQR)	23.0 (9.0–58.0)	43.5 (12.8–86.3)
Antifungal prophylaxis		
Micafungin	2 (13)	1 (7)
Posaconazole	1 (7)	2 (14)
Fluconazole	2 (13)	2 (14)
No antifungal prophylaxis	10 (67)	9 (64)
Antifungal agent^g^		
Voriconazole	11 (65)	11 (73)
Amphotericin B	5 (29)	1 (7)
Echinocandin	1 (6)	1 (7)
Others	0 (0)	2 (13)
30-day mortality	5 (29)	3 (20)

*IQR* interquartile range

^a^From September 2017 – October 2017, during demolition and excavation works

^b^From November 2017 – February 2018, during framing, interior designing, plumbing, and finishing works

^c^1 chronic myeloid leukemia, 6 aplastic anemia, 1 multiple myeloma, 1 hemophagocytic lymphohistiocytosis, 1 neuroblastoma

^d^Absolute neutrophil count < 500/mm^3^ for more than 10 days

^e^ > 0.3 mg/kg/day prednisolone for more than 3 weeks

^f^Excluded patients who did not undergo appropriate tests

^g^Antifungal agent that was used for the treatment of current invasive aspergillosis for over 75% of the total duration

### Air sampling for environmental surveillance

**Fig. 1:**
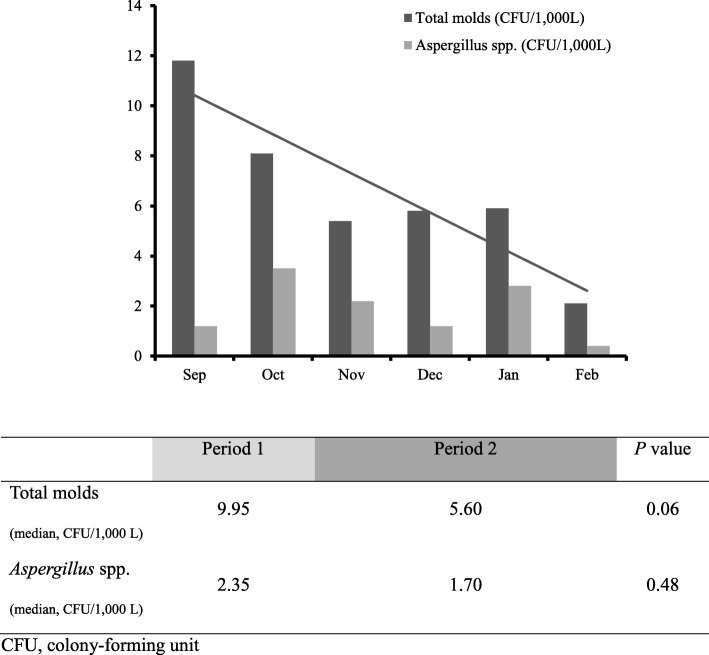
Airborne fungal spore levels in three hematologic wards during a 6 month construction period. CFU, colony-forming unit

Figure 1 shows the airborne fungal spore levels (both total mold and *Aspergillus* spp.) in three hematologic wards during the 6 month construction period. Compared with that of period 1 (9.95 CFU/1000 L), the total mold spore level tended to be lower in period 2 (5.60 CFU/1000 L), which is depicted by the linear regression line of best fit (*p* = 0.06). Of the total mold spores, those of *Penicillium* spp. were the most common. *Aspergillus* spp. spore levels were also lower in period 2 (1.70 CFU/1000 L) than in period 1 (2.35 CFU/1000 L), although the difference was not significant (*p* = 0.48). Ward 84 had the highest total mold and *Aspergillus* spp. spore levels at 12.12 and 3.86 CFU/1000 L, respectively (*p* = 0.01 and 0.03).

### Incidence of invasive aspergillosis

**Fig. 2:**
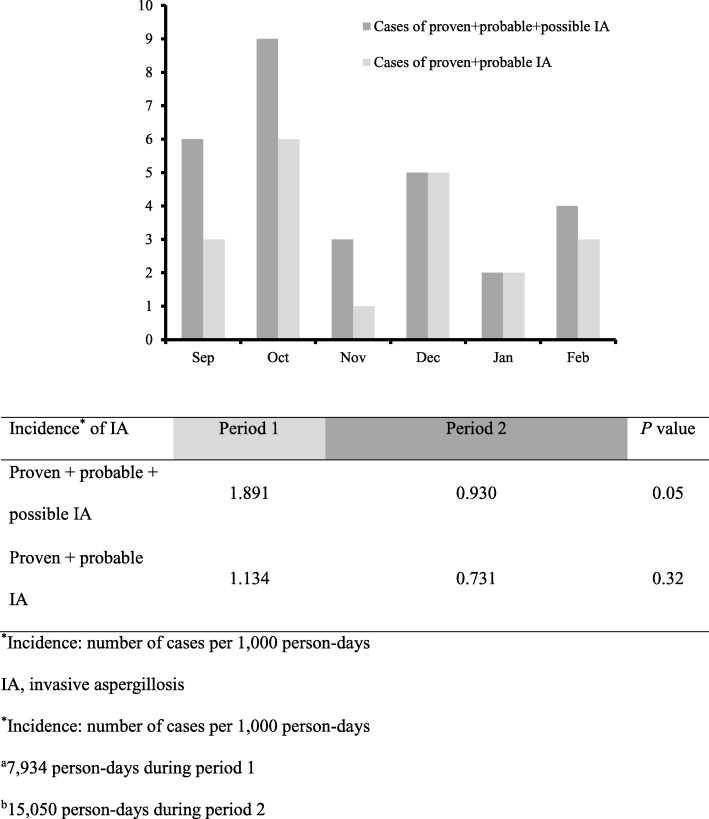
Incidence of invasive aspergillosis in three hematologic wards during a 6 month constructionperiod. ^*^Incidence: number of cases per 1000 person-days. IA, invasive aspergillosis. ^*^Incidence: number of cases per 1000 person-days. ^a^7,934 person-days during period 1. ^b^15,050 person-days during period 2

Figure 2 shows the incidence of IA in the three hematologic wards during the 6 month construction period. The incidence of all IA by the EORTC/MSG criteria was also higher in period 1 than in period 2 (1.891 vs. 0.930 per 1000 person-days, *p* = 0.05). Thus, the incidence of IA was higher in period 1 when airborne fungal spore levels tended to be higher. In the entire study period, of the three wards, ward 84 had the highest incidence of total IA (1.996 per 1000 person-days, *p* = 0.049).

## Discussion

We investigated the relationship between airborne fungal contamination and the incidence of IA during construction periods in a tertiary care hospital. Construction period 1 (demolition and excavation) tended to have higher levels of airborne spore levels than in construction period 2 (framing, interior designing, plumbing, and finishing), both in terms of total mold spores (9.95 vs. 5.60 CFU/1000 L) and *Aspergillus* spp. spores (2.35 vs. 1.70 CFU/1000 L). The total incidence rate of IA was significantly higher in period 1 than in period 2 (1.891 vs. 0.930/1000 person-days).

The incidence of IA was higher in period 1, when airborne fungal spore levels tended to be higher, than in period 2. All demolition and excavation works were performed in period 1; although one study showed that demolition of a hospital building significantly increased fungal spore levels in hospital external and non-protected internal air [16], the correlation between specific construction types and airborne fungal contamination and the incidence of IA has not been well-studied [8]. According to our data, demolition and excavation works seemed to have affected the airborne total mold and *Aspergillus* spp. spore levels and may have contributed to the increased incidence of IA. This finding may be explained by the risk of large amounts of fungal spore being dispersed during demolition and excavation works than during other types of construction works. The differences in spore levels between periods 1 and 2 were more pronounced for total mold spores than for those of *Aspergillus* spp. Although the pathogen of IA is *Aspergillus* spp., high total mold spore levels may indicate ineffective air filtration and/or the existence of conditions that favor the settling of molds, including *Aspergillus* spp. [9]. Therefore, total mold spore level could serve as an indirect marker of air contamination that may be associated with the spore levels of other pathogens.

Construction works are regarded as a possible source of IA outbreaks in hospitals [8–11]. For this reason, the Centers for Disease Control and Prevention recommend active surveillance for airborne fungal infection cases in high-risk patients in hospitals during constructions [6]. However, there are some problems in conducting active surveillance in patients. First, the diagnostic performance of serum galactomannan or fungal culture from clinical specimens is relatively low [17]. Some IA patients may go undiagnosed if the diagnosis is made based on the EORTC/MSG criteria due to the low sensitivities of the diagnostic tests. There is also the possibility of false positivity of serum galactomannan or colonization of the respiratory tract of patients by *Aspergillus* spp. Second, there are problems defining hospital-acquired IA because the incubation period of IA is yet to be clearly defined [7, 15, 18]. In this context, it is possible that patients who were exposed in period 1 could have developed IA in period 2 and IA in period 1 could reflect exposure during the preceding weeks of hospitalization.

To prevent IA during construction periods, the Centers for Disease Control and Prevention guideline recommends implementation of infection-control measures, such as sealing windows and specific ventilation systems, based on infection-control risk assessment [6]. However, studies on the efficacy of HEPA filters for controlling air contamination have shown conflicting results [9, 10, 19]. In our current study, ward 84 without any specific ventilation system had the highest levels of both total mold and *Aspergillus* spp. spores, and the incidence of IA was also higher than in other wards. Although we did not describe the exact efficacy of the HEPA filters, our data show that fungal contamination tended to be higher in conventional wards and lower in the wards equipped with HEPA filters.

The recommendation of routine environmental surveillance before, during, or after construction is still under debate. There are several issues with environmental surveillance by air sampling. Importantly, there is no gold standard for sampling air for environmental surveillance, such as the appropriate volume, number, and location for collection. In addition, there is no consensus on the cut-off value for designating fungal spore level as either safe or dangerous. According to a previous study, airborne fungal contamination during IA outbreaks in several hospitals varied widely from 0 to more than 100 spores/m^3^ [11]. Nevertheless, based on our current results, we suggest that airborne fungal spore levels be monitored during construction in hospitals with immunocompromised patients, and that further studies are needed to set a cut-off value for a “dangerous” level of fungal spore contamination.

This study has several limitations. First, we performed air sampling only three times on a given day on a monthly basis, and the spore levels of one day may not be representative of the spore levels of the month as a whole. However, standardized methods of air sampling such as determining where, how often, or how much air should be sampled have not been established yet. Thus, further studies are needed in this area. Second, we could not account for the various factors that may affect the spore levels of airborne molds, such as season, weather, and ventilation system, which may ultimately act as potential confounders for the difference in IA incidence. Because our data could not be compared with the baseline data on airborne fungal contamination prior to construction, it was difficult to evaluate the impact of construction works on airborne fungal contamination independently. In general, the spore levels of airborne molds were significantly higher during summer than during the other seasons [20–23]. However, *Aspergillus* spp. spore concentration did not correlate with various meteorological data, such as temperature, precipitation, and humidity [13, 20, 22], although there were some reports about seasonal variation of *Aspergillus* spp. spore levels [24, 25]. Therefore, it is not yet clear how meteorological variation affects *Aspergillus* spp. spore levels, not total molds spore levels, and the incidence of IA. It is worth noting that we have prospectively monitored weekly cases of *Aspergillus* spp. isolates from clinical specimens from January 2016 to December 2018 (Additional file 1: Figure S1). There were no seasonal variations in *Aspergillus* spp. isolation from clinical specimens. A previous study revealed that there was a significant correlation between *Aspergillus* spp. colonization from respiratory tract and airborne fungal contamination of patients’ rooms on multivariate analysis [26]. Taken together, it is less likely that increased airborne spore levels and incidence of IA during the construction period might be caused by seasonal variation, although the absence of baseline year-round air sampling before construction work makes it difficult to arrive at a firm conclusion on seasonal variations. Further studies are needed to clarify this area. Third, there might be several variables affecting the risk of IA other than construction works. Particularly, different clinical characteristics of patients who were exposed during period 1 and period 2 might account for the differences in the incidence of IA. However, there were no significant differences in the duration of neutropenia or type of hematologic diseases except for acute myeloid leukemia among patients admitted between periods 1 and 2 (Additional file 1: Table S1). Fourth, there might be a learning effect over time and improved diagnostic technology for diagnosing IA. However, because we analyzed data during only 6 months, and there was no significant change or development in the treatment strategies, diagnostic criteria or technology, this effect seemed to be less influential. Fifth, although we found a relationship between the incidence of IA and total mold spore levels during construction works, we could not reveal the direct relationship between the incidence of IA and *Aspergillus* spp. spore levels. However, considering that identification of *Aspergillus* spp. is usually difficult, total mold spores could be a surrogate marker of air contamination contributing to IA. Finally, we did not perform a genomic analysis of the *Aspergillus* spp. isolated from the clinical and air samples. It remains a difficult task to identify a direct epidemiological linkage between environmental and clinical fungal isolates, with two recent studies showing conflicting results on the correlation between *A. fumigatus* spore levels in the air and the incidence of IA during constructions in hospitals [12, 13]. Further studies are needed to clarify the genomic correlation between environmental and clinical fungal isolates.

## Conclusions

We found that the incidence of IA was significantly higher during heavy hospital construction works such as demolition and excavation, during which airborne fungal spore levels also tended to be higher. We recommend that airborne fungal spore levels be monitored during construction periods in hospitals with immunocompromised patients. Subsequently, the effect of airborne fungal spore level monitoring in reducing hospital-acquired IA should be evaluated.

## Additional file

Additional file 1: **Table S1.** Baseline characteristics of admitted patients during periods 1 and 2. **Figure S1.** Cases of *Aspergillus* spp. isolation from clinical specimens from January 2016 to December 2018. (ZIP 671 kb)

## Data Availability

The datasets used and/or analyzed during the current study are available from the corresponding author on reasonable request.

## Abbreviations

IA: Invasive aspergillosis
EORTC/MSG: European Organization for Research and Treatment of Cancer/Mycosis Study Group
HEPA: High-efficiency particulate air
HSCT: Hematopoietic stem cell transplantation
CFU: Colony-forming units
