# Supplementary material for: Airborne fungal spores and invasive aspergillosis in hematologic units in a tertiary hospital during construction: a prospective cohort study
Source: Antimicrob Resist Infect Control. 2019 May 29;8:88. doi: 10.1186/s13756-019-0543-1 (PMC6542016; doi:10.1186/s13756-019-0543-1)

**Supplemental Table 1. Baseline characteristics of admitted patients during periods 1 and 2**

|  | **Period 1 (n=57)^a^** | **Period 2 (n=211)^a^** | ***P* value** |
| --- | --- | --- | --- |
| **Age, median years (IQR)** | 23 (7-56) | 36 (9-56) | 0.20 |
| **Male gender, no. (%)** | 36 (63) | 115 (55) | 0.24 |
| **Underlying hematologic disease, no.(%)** |  |  |  |
| Acute myeloid leukemia | 10 (18) | 68 (32) | 0.03 |
| Acute lymphoid leukemia | 8 (14) | 30 (14) | 0.97 |
| Myelodysplatic syndrome | 6 (27) | 16 (8) | 0.43 |
| Lymphoma | 5 (9) | 23 (11) | 0.64 |
| Others^c^ | 27 (47) | 74 (35) | 0.09 |
| **Underlying condition** |  |  |  |
| Neutropenia | 30 (53) | 135 (64) | 0.12 |
| Hematopoietic stem cell transplant | 22 (39) | 63 (30) | 0.21 |
| Steroid use | 9 (16) | 19 (9) | 0.14 |
| **Antifungal prophylaxis** | 14 (25) | 50 (24) | 0.89 |
| Micafungin | 9 (16) | 29 (14) |  |
| Posaconazole | 2 (4) | 19 (9) |  |
| Fluconazole | 3 (5) | 2 (1) |  |

^a^ All hospitalized patients (n=268) in three hematologic wards in period 1 and period 2, respectively, except those who developed IA (n=29) and were admitted both in period 1 and period 2 (n=159).

**Supplemental Fig. 1. Cases of *Aspergillus* spp. isolation from clinical specimens from January 2016 to December 2018**


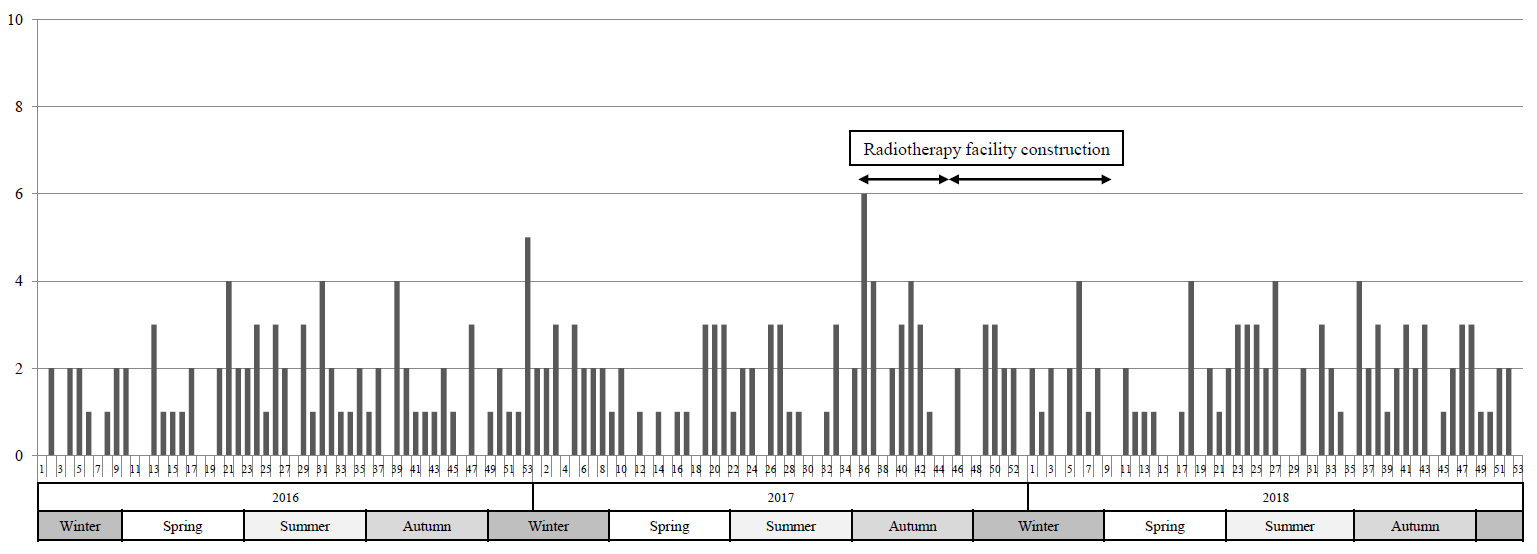

Supplement: Supplementary file 1 — Table S1. Baseline characteristics of admitted patients during periods 1 and 2. Figure S1. Cases of Aspergillus spp. isolation from clinical specimens from January 2016 to December 2018. (ZIP 671 kb) [file 13756_2019_543_MOESM1_ESM.zip › Supplemental Table & Figure.docx]
