# Supplementary figures and images for: Airborne fungal spores and invasive aspergillosis in hematologic units in a tertiary hospital during construction: a prospective cohort study
Source: Antimicrob Resist Infect Control. 2019 May 29;8:88. doi: 10.1186/s13756-019-0543-1 (PMC6542016; doi:10.1186/s13756-019-0543-1)

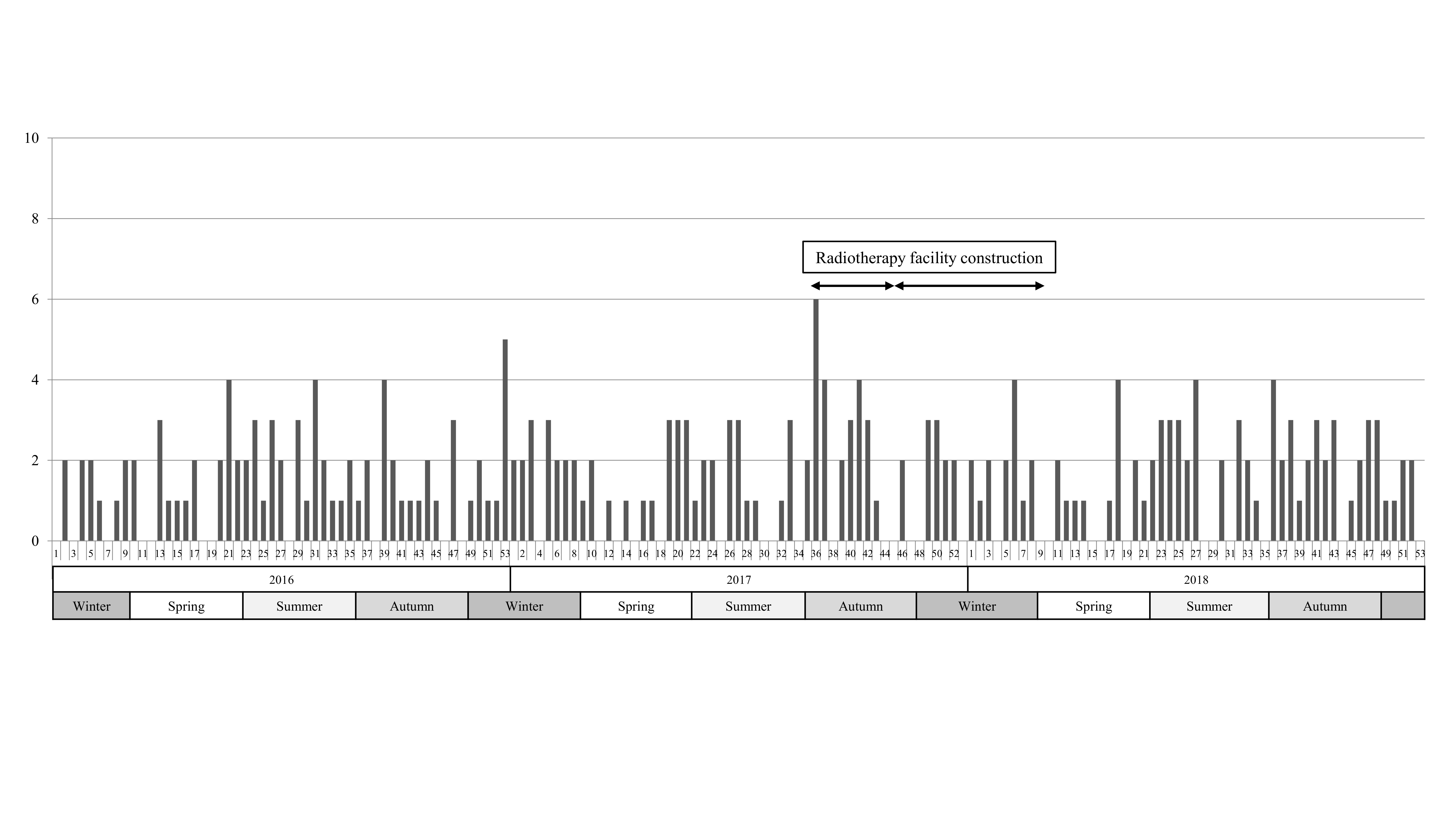

Supplement: Supplementary file 1 — Table S1. Baseline characteristics of admitted patients during periods 1 and 2. Figure S1. Cases of Aspergillus spp. isolation from clinical specimens from January 2016 to December 2018. (ZIP 671 kb) [file 13756_2019_543_MOESM1_ESM.zip › Supplemental Fig1.tif]
